# Unveiling Current Guanaco Distribution in Chile Based upon Niche Structure of Phylogeographic Lineages: Andean Puna to Subpolar Forests

**DOI:** 10.1371/journal.pone.0078894

**Published:** 2013-11-12

**Authors:** Benito A. González, Horacio Samaniego, Juan Carlos Marín, Cristián F. Estades

**Affiliations:** 1 Laboratorio de Ecología de Vida Silvestre, Facultad de Ciencias Forestales y de la Conservación de la Naturaleza, Universidad de Chile, Santiago, Chile; 2 Laboratorio de Ecoinformática, Instituto de Conservación, Biodiversidad y Territorio, Universidad Austral de Chile, Valdivia, Chile; 3 Centro de Investigación en Complejidad Social, Universidad del Desarrollo, Santiago, Chile; 4 Center for Non Linear Studies, Los Alamos National Laboratory, Los Alamos, New Mexico, United States of America; 5 Laboratorio de Genómica y Biodiversidad, Departamento de Ciencias Básicas, Facultad de Ciencias, Universidad del Bío-Bío, Chillán, Chile; Ecole Normale Supérieure de Lyon, France

## Abstract

Niche description and differentiation at broad geographic scales have been recent major topics in ecology and evolution. Describing the environmental niche structure of sister taxa with known evolutionary trajectories stands out as a useful exercise in understanding niche requirements. Here we model the environmental niche structure and distribution of the recently resolved phylogeography of guanaco (*Lama guanicoe*) lineages on the western slope of the southern Andes. Using a maximum entropy framework, field data, and information on climate, topography, human density, and vegetation cover, we identify differences between the two subspecies (*L.g.cacsilensis*, *L.g.guanicoe*) and their intermediate-hybrid lineage, that most likely determine the distribution of this species. While aridity seems to be a major factor influencing the distribution at the species-level (annual precipitation <900 mm), we also document important differences in niche specificity for each subspecies, where distribution of Northern lineage is explained mainly by elevation (mean = 3,413 m) and precipitation seasonality (mean = 161 mm), hybrid lineage by annual precipitation (mean = 139 mm), and Southern subspecies by annual precipitation (mean = 553 mm), precipitation seasonality (mean = 21 mm) and grass cover (mean = 8.2%). Among lineages, we detected low levels of niche overlap: ***I*** (Similarity Index) = 0.06 and ***D*** (Schoener’s Similarity Index) = 0.01; and higher levels when comparing Northern and Southern subspecies with hybrids lineage (***I*** = 0.32-0.10 and ***D*** = 0.12-0.03, respectively). This suggests that important ecological and/or evolutionary processes are shaping the niche of guanacos in Chile, producing discrepancies when comparing range distribution at the species-level (81,756 km^2^) with lineages-level (65,321 km^2^). The subspecies-specific description of niche structure is provided here based upon detailed spatial distribution of the lineages of guanacos in Chile. Such description provides a scientific tool to further develop large scale plans for habitat conservation and preservation of intraspecific genetic variability for this far ranging South American camelid, which inhabits a diversity of ecoregion types from Andean puna to subpolar forests.

## Introduction

Geographic distribution range is an emerging feature of species’ hierarchical selection of their habitat [1]. Despite its importance for species persistence across the landscape, the exact distribution within the environment is often difficult to ascertain, and although a large body of work exists on the subject, the tenets of niche structure are still poorly understood [2]. Such complications for estimating species distribution most likely stem from the myriad of interactions between two major influences on a species: its innate characteristics (e.g. evolutionary history, specialization, physiological tolerance, and resource requirements) and its environment (e.g. competitive interactions, predator-prey relationships, and resources availability) [3]–[5].

A species’ niche is ultimately a mapping or description of its whole environment from birth until death, and can thus be thought as an abstraction of multi-dimensional forces in the environment acting upon the persistence of individuals [6]–[8]. Thus, the development of models describing the niche is a valuable tool for not only understanding how a species distributes itself across human modified landscapes, but for understanding and predicting how a species will cope with and be impacted by current shifts in climate [2], [8]–[11]. For instance, niche models have been used in several different scenarios to predict species presence when information is spatially-incomplete; to predict temporal changes in environmental variables (temporal extrapolation), or to acquire information on the biological mechanisms explaining potential distribution [1], [12]–[14]. As such, niche models have become fundamental in several areas of conservation biology, including the design of local nature reserves, the estimation of potential habitats of endangered or invasive species, and even the identification of subtle but important changes in the distribution of taxa associated with climate change [15]–[17].

The guanaco (*Lama guanicoe*) is a South American wild, undomesticated and protected ungulate found from sea level to over 4500 m [18]. Guanaco habitat is characterized by a highly seasonal climate, with dry and occasionally snowy winters, subjected to moderate to high intensity winds, and variable rainfall. All these factors usually generate high evapotranspiration and, when arid conditions prevail, are responsible for low productivity [19]. Guanaco dwell in four of the ten major habitats described in South America [18]: (i) Desert and Xeric Shrublands; (ii) Montane Grasslands; (iii) Grasslands, Shrublands and Savannas; and (iv) Temperate Forest. In Chile, guanaco distribution has been mostly discussed in qualitative terms [18]–[19]. Given the large and conspicuous size of the guanaco (∼120 kg; [18], it is posited that current geographic range extends from Putre (18.5°S latitude) to Navarino Island, Chile (55°S latitude), with discontinuities associated with human presence [20]. However, contemporary guanaco distribution has not been systematically evaluated in a spatial explicit context [21]. Moreover, environmental niche factors associated to guanaco distribution have not been modeled, nor reviewed.

Recent work on the evolutionary and geographic distribution history of the guanaco in Chile, documented by mitochondrial and nuclear genetic markers, show the existence of two major evolutionary lineages or subspecies (corresponding to *Lama guanicoe cacsilensis* in the north and *L. g. guanicoe* in the south) [18], [22], [23]. These studies suggest that *L. g. guanicoe* may have expanded southward during the late Pleistocene and early Holocene from subtropical refugia. This lineage is currently found in Bolivia, northern Argentina, central Chile as well as in continental and insular Patagonia. The other group (*L. g. cacsilensis*) appears to have been confined to the coastal desert of Peru and northern Chile, suggesting the existence of different niche structures between groups. Moreover, a hybrid range has been detected, where individuals share genetic characteristics of both lineages, expanding the coverage of guanaco distribution and possibly the occupation of a new niche [23].

Here we provide a systematic analysis of species range using a maximum entropy framework to unveil and understand evolutionary patterns of guanaco distribution. Towards this goal, we analyze the geographic distribution of the guanaco in Chile, based upon its two recently detected phylogeographic lineages and its intermediate hybrid [23]. The heterogeneity of ecosystems occupied by guanacos makes it an ideal study-system-species to understand how spatial distribution patterns relate to phylogeographic processes and the possibility of being predicted based upon broad scale environmental factors.

## Materials and Methods

### Ethics Statement

The present study did not require the capture or handling of animals from any protected or endangered guanaco populations, which is classified as “Least Concern” by the Red List, IUCN. Data for this study is based upon indirect information or from direct observations by the authors. While special permits are not required for observational studies under Chilean law, access to protected areas to conduct such observations was appropriately granted to the authors of this study, either by private land owners or by the Chilean Corporación Nacional Forestal (CONAF), where required. See acknowledgment section for a complete list of collaborators who granted access and provided information for the current study.

### Data Sources

**Figure 1:**
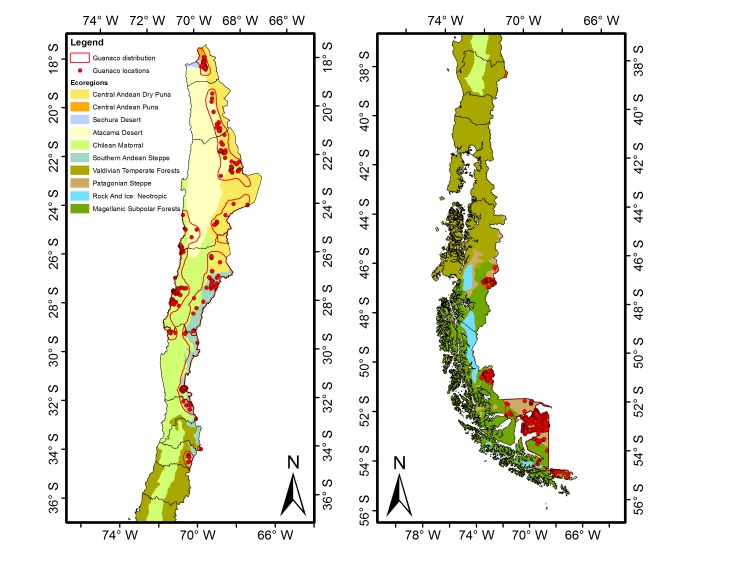
Locations of guanacos in Chile used for environmental niche modeling (red dots). Areas in color indicate ecoregions according to [63], and red line indicates the edge of the guanaco distribution according to [44].

We compiled a dataset of 2,962 guanaco locations in Chile between 2000 and 2011 (Dataset S1). The conspicuousness of the species and the size of the dataset, suggest that the current distribution of the species has been represented with a reasonable level of completeness throughout the country (Figure 1). Observations consisted of direct sightings of animals and indirect evidence of guanaco presence at each particular location (i.e. feces collection, tissue collections from live and dead animals, carcasses) made by the authors of this study and collaborators (see acknowledgements). Each location was assigned to 525, 3×3 km cells which roughly correspond to the minimum average home range size detected for a sedentary population of guanacos [24].

### Variable Selection

Predictor variables were selected a priori based on natural history knowledge of the guanaco and its relationship to climate, topography, vegetation, and anthropogenic pressures. Such decisions follow the need to avoid data reduction procedures known to generate new sets of variables with questionable interpretation, and low amenability for comparison in an evolutionary context given the spatial and temporal scale used and the correlation among explanatory variables [7], [25]. All data were scaled to a 9 km^2^ resolution using an average rule. A total of four variables were used among the 19 bioclimatic variables provided by the WorldClim database [26]. Chosen variables included were: annual mean temperature (BIO1), temperature seasonality (BIO4), annual precipitation (BIO12), precipitation seasonality (BIO15), and the average yearly minimum and maximum temperatures. Roughness, aspect, slope, and elevation were obtained from the Shuttle Radar Topographic Mission (SRTM) [27]. Enhanced Vegetation Index (EVI) was used as a proxy for vegetation productivity. Unlike the Normalized Difference Vegetation Index (NDVI), which is sensitive to the presence of chlorophyll, EVI considers the variations in canopy structure [28]. Also, land cover data (1 km resolution) from the Global Land Cover, provided information relative to land cover using the following classes: bare soil, grasses, and shrubs/trees [29]. Finally, anthropogenic influence was considered through the 2008 human population density (number of inhabitants/km^2^) dataset from the Socioeconomic Data and Applications Center [30].

### Statistical Models, Validation, and Variable Contributions

Geographic ranges were estimated using maximum entropy (Maxent) [31]. Maxent is a machine learning algorithm that uses the multivariate distribution of suitable habitat conditions inferred from presence records to generate a probability surface of species presence as close to a uniform distribution as possible, given the constraints imposed by the expected value of the distribution of the environmental features at each location were the species has been recorded [7], [11], [12], [32], [33]. Hence, Maxent is based on the assumption that each pixel or cell within the study area has a probability distribution, where habitat is not available (*p = 0*), up to a complete probability (*p = 1.0*) defining the best and most suitable habitat for the species. Pixels with a geo-referenced record constitute a sampled area, and the environmental variables associated with that specific cell (climatic, physiographic, etc.) are the environmental characteristics explaining the occurrence within that particular pixel [31]. One of the major constraints for the remaining cells (that do not contain geo-referenced records) is that the expected value of each variable should match with the empirical average obtained from the sampled areas. This results in a probability distribution that maximizes the entropy of the unknown (species presence in the given the environment) [31]. In practical terms, this implies that the model considers everything that is known, but carefully avoids assuming anything that is unknown [34].

As an input to Maxent, locations of guanaco presence were randomly divided into two sub-samples for cross-validation. A first dataset with 20% of our guanaco locations were used as a training set and the remaining 80% to validate the resulting model. This partition choice arises as function of the number of variables employed in our models following [11]. 50 models were run with 500 repetitions. The adjustment measure implemented by Maxent is the area under the curve (AUC) from the ROC (Receiver Operating Characteristic plot). Such adjustment-measures compare model sensitivity to specificity, i.e. the proportion of points considered to the ratio of absences considered outside the model. AUC scores range from 0 (worse than randomly generated) to 1, when the model has a perfect discrimination [35]. Models with AUC score greater or equal to 0.7 are considered useful [36].

Maxent estimates the relative contribution of all environmental variables in the model and delivers a jackknife analysis for each [31], [33]. Response curves were examined for each environmental variable involved in the model and predicted changes when a specific environmental factor was altered, while the remaining variables were kept at their average value. Such methodology allowed for the evaluation of the marginal effect of changing one variable at the time in the model.

### Comparing Distributions between Phylogeographic Lineages and Species Range

The distribution of the species was compared to the joint distribution of the two lineages of guanacos and that of their hybrid zone. The probability map was converted to a binary map of presence-absence using a 10% threshold based on the logistic model (∼0.25 of predicted habitat suitability). This was done after the exclusion of the 10% of the extreme observations, as they may represent pixels with transient populations or unusual environmental conditions [37]. Finally, overlaps between lineage models (niche) and distributions range were estimated following [38], [39].

## Results

### Prediction and Comparison of Species and Subspecies Distribution Ranges

All maximum entropy models had high AUC values. The model for species level showed an AUC = 0.921±0.023. AUC for the Northern subspecies (*L. g. cacsilensis*) in the north was 0.976±0.019, whereas the Southern subspecies (*L. g. guanicoe*) in the south was 0.971±0.011. The hybridization zone between the two showed values of AUC = 0.978±0.018.

**Figure 2:**
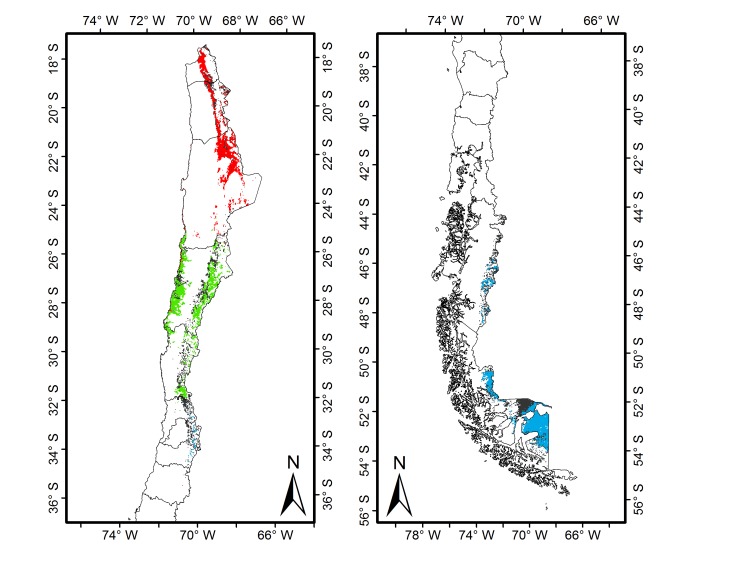
Map of geographic distribution range of guanaco linages in Chile. *L. g. cacsilensis*, hybrid lineage and *L. g. guanicoe* in red, green and blue, respectively. Disagreement area between species-level and intraspecific-level models are shown in black.

Estimated distributional areas were the following: the entire species in Chile was 81,756 km^2^, whereas for the Northern subspecies it was 21,497 km^2^, Southern subspecies 20,767 km^2^, and the hybridization zone between both taxa was of 23,481 km^2^, combining a total surface of 65,321 km^2^ (maps in Figures S1, S2, S3, S4 and S5). Common area between the species-level and intraspecific level models was 47,740 km^2^, whereas the area of disagreement was higher when contrasting the species-level model to the intraspecific level model (34,020 km^2^ vs. 17,577 km^2^, Figure 2). This indicates that the species-level model generated a wider projection area than the subspecies-level model; however, the latter included areas not considered by the species model. Therefore, to elaborate a potential species distribution map for Chile, a joint model was generated with a total area of 99,337 km^2^, which roughly corresponds to 13.1% of the total country surface area. Overlap was marginal between each subspecies and the hybrid range with 0.014 and 0.0004% for Northern and Southern, respectively [39].

### Environmental Niche

Species and subspecies distribution models gave greater importance to climate variables than to any other environmental variable (Table 1, Table S1), confirming the hypothesis that climatic variables significantly contribute to the occurrence of guanacos in Chile. Variables that contributed the most to explain the distribution of guanacos were annual rainfall and annual average minimum temperatures, with a join contribution of 57% to the model. Elevation and herbaceous cover contributed 23%. Accordingly, the Northern subspecies, which is restricted to Chile’s arid zone, exhibited a similar pattern when climatic variables were also selected. Rainfall seasonality and annual average maximum temperature contributed 45% and elevation only 34%. Similarly, the predicted range of the Andean and Patagonia distribution of the Southern subspecies was mostly explained by climate, in which average annual temperature, annual rainfall, and rainfall seasonality contributed 64%. Herbaceous cover contributed 22% to the model. Across the hybridization area, north of Chile’s Mediterranean zone and including the coastal and Andean populations, the potential distribution of guanaco populations can again be mostly explained by climatic factors. Temperature seasonality, annual rainfall, and rainfall seasonality contributed 69% to the model, and only one of the vegetation variables - EVI - provided ∼8%.

**Table 1:** Relative percent contributions of the environmental variables to environmental niche models of guanaco linages in Chile. Bold numbers indicate higher scores.

Variables	*Lama guanicoe*	*L. g. cacsilensis*(Northern subspecies)	Intermediate-Hybrid linage	*L. g. guanicoe* (Southern subspecies)
**Bioclimatic**				
Annual mean temperature	3.8	2.3	0.9	**12.9**
Temperature Seasonality	1.8	6.0	**11.6**	3.8
Average annual minimum temperatures	**18.5**	0.2	6.3	3.2
Average annual maximum temperatures	0.4	**12.6**	0.0	1.7
Annual precipitation	**38.3**	2.6	**43.6**	**26.8**
Precipitation seasonality	7.0	**32.8**	**13.9**	**24.5**
**Topographic**				
Elevation	**10.3**	**34.6**	6.1	1.4
Slope	0.3	1.8	0.6	0.6
Aspect	1.0	1.1	1.4	0.2
Roughness	0.8	0.9	0.7	0.6
**Vegetation**				
EVI (Enhanced vegetation Index)	1.8	0.9	**7.7**	0.7
Bare soil cover (%)	0.4	0.9	1.0	0.5
Grass cover (%)	**12.4**	2.7	1.7	**22.5**
Shrubs and trees cover (%)	0.7	0.1	3.5	0.2
**Anthropic**				
Population density (people/9 km^2^, 2008)	2.5	0.5	0.9	0.5

Comparison of niche breadth and overlap are shown in Tables 2 and 3, respectively [40]. While niche breadth is relatively similar among lineages, niche overlap is consistently smaller between the Southern and all other lineages for all indices. This becomes even more obvious when evaluating range overlap using the common threshold of 25% suitability, in which the Southern subspecies stands as a geographically isolated lineage given the fact that all analyses were restricted to the geopolitical boundaries of Chile.

**Table 2:** Environmental niche modeling analysis of niche breadth for guanaco in Chile.

	Niche breadth (entropy)
***L. g. cacsilensis*** (Northern subspecies)	0.81
**Intermediate-Hybrid lineage**	0.82
***L. g. guanicoe*** (Southern subspecies)	0.79
***L. guanicoe*** (all lineages)	0.90

**Table 3:** Niche overlap among guanaco lineages.

	Niche overlap index			Range overlap
Compared lineages	*I*	*D*	Relative Rank	
Northern - All	0.57	0.29	0.73	0.46
Northern - Hybrid	0.32	0.12	0.81	0.01
Northern - Southern	0.06	0.01	0.45	0.00
Southern - Hybrid	0.10	0.03	0.46	0.00
Southern - All	0.70	0.44	0.64	0.96
Hybrid - All	0.59	0.33	0.71	0.42

Similarity (***I***), Schoener’s distance (***D***), and **Relative Rank index** indicate similarity between niche overlap. **Range overlap** is based on ranges where 0.25 threshold of suitability is assumed.

## Discussion

This study identifies some of the factors that determine the environmental niche structure and geographic distribution range of guanaco subspecies in Chile. Using knowledge of climate, geography, human density, vegetation cover and the recently unveiled phylogeography of guanaco [23], we have attempted to understand the most important environmental parameters likely to determine the distribution of this South American camelid. In spite of the increasing knowledge on several areas of the guanaco ecology [41], this is, to our knowledge, the first attempt to quantify the ecological niche of the guanaco and its phylogenetic lineages [23]. Important contributions on the spatial distribution of guanaco have explained species occurrence at smaller geographic scale in relation to intra-annual environmental variations [21], [42], [43]. This highly detailed spatial description of the guanaco relative to its environmental correlates and genetic diversity in Chile, further contributes to provide scientific tools to develop large scale conservation plans for habitat management that considers genetic variability [17 20, 21, 44].

Some of the environmental variables that contributed very little to the model were related to cover type, and anthropogenic factors. Unexpectedly, vegetation structure and variables associated with topography, specifically roughness and slope, were not relevant to the model. The literature indicates that these factors are relevant for guanaco presence, which in some cases has been shown to prefer mountainous areas of medium to high slopes, with herbaceous and shrubby vegetation cover [42]–[44]; however, this was not reflected using our large-scale analysis. Finally and contrary to our expectations, the anthropic factor was not relevant for the determination of the guanaco’s potential niche.

It is largely accepted that genetic structure and history are important considerations for the long-term and successful implementation of modern conservation plans. We show that while guanaco lineages share some similarities in their niche structure, important differences exist (Table 1). In fact, different models accurately predict guanaco distribution and do this at different phylogenetic scales (cf. species vs. subspecies). One potential explanation is the existence of particular tendencies among lineages to retain ecological preferences over time, ie. niche conservatism [45]–[47]. Alternative processes of niche divergence or neutral genetic processes could also be at play [48], [49]. However, disentangle the exact mechanism would entail the analysis of guanaco occurrence beyond administrative boundaries of Chile and across its full distribution range, including Peru, Bolivia and Argentina. We are currently working on such dataset compilation.

Other environmental variables became important at the species and subspecies level. We found no indications of a larger niche breadth for lineages bearing shorter evolutionary histories and occupation of extant habitats. This is apparent by looking at the similarities in niche breadth [50] and limited niche overlap between northern and southern lineages (Table 3). Finally, the lack of geographic range overlap between lineages (Table 3) strongly suggests the existence of fundamental differences between environmental requirements for the two subspecies and the hybrid lineage.

From a biogeographical perspective, some researchers have associated the guanacos only to xeric environments and have even classified them as *stenohydropedomorphic*, that is of low tolerance to wet soils [51], [52]. We show that, in fact, low annual rainfall is strongly associated with guanaco distribution throughout the species’ range and includes the northern hyper-arid Atacama Desert, the Cordillera, and Southern Patagonia including Tierra del Fuego and Navarino Island in Chile. While this indicates that guanaco subspecies currently presents an affinity for such environments, the quantitative analysis presented here suggests that the broad generalization proposed by [52] to current distributional patterns of Southamerican camelids based on associations to the Arid Diagonal, falls short to explain current guanaco distribution given the large dissimilarities among phylogeographic lineages. The phylogeographic history of each lineage (*L.g. cacsilensis* vs. *L.g. guanicoe*) indicates that the possible biogeographic barrier [53] between lineages was colonized by a successful hybrid lineage –most likely in several attempts as different shared haplotypes exist between lineages [23]. Hence, explaining the asymmetrically-shared niche structures between both subspecies, and possibly limiting their differentiation due to trailing gene flow between taxa [23].

Models generated at both the species and subspecies level explain how the relationship between environmental variables and some of the anatomical, physiological, and reproductive adaptations allowed the guanaco to be efficient in arid habitats [18]. For example, the presence of a thin wooly insulation coat in guanaco that allows it to withstand extreme temperatures, the capability to adjust timing of parturition with benign environmental conditions [18], [41], as well as an efficient digestive system to absorb adequate nutrition from medium and low quality forage. Such type of vegetation is typical in arid environments and is characterized by seasonal growth [18], [19]. This may contribute to understand the correlation between guanaco and the distribution of micro- and meso-thermal graminoids of arid systems that are highly resistant to herbivores [51]. While such features were evident when evaluating the model for the entire species, it was more apparent for the *L. g. guanicoe* lineage, where the model selected herbaceous cover with approximate average values between 60 and 70%.

At the country and species level, our environmental niche model shows three areas of low habitat suitability that correlates with large biogeographic barriers: (1) Pre-Altiplano Andean zone, running from the border with Peru (18**°**S latitude) to the end of the Altiplano plateau in Chile 24.5**°**S; (2). The central zone, flanked by the arid coast running continuously between 24.7**°**S and 29.5**°**S, and the western Andes slope (24.5**°**S to 35.3**°**S); and (3) Patagonia, which –in Chile–extends discontinuously from the Araucania region (35.3**°**S) to the southern tip of the continent. Between the Andean highlands and the central region of Chile, the presence of the Atacama Desert is likely to have created an environmental barrier disrupting guanaco distribution. As suggested by [54] for the biodiversity in Chile, the Arid Diagonal has specifically limited the distribution of several mammals in northern Chile such as vicuña (*Vicugna vicugna)*. Between the coast and the Andean mountains of the arid north of Chile, approximately from parallel 24.6**°**S to 29.5**°**S, there is a central strip with low probability of occurrence of guanacos, probably due to the extremely dry conditions of the area. However, the suitability for guanaco (and other species) is strongly improved during climatic events not captured by our models (i.e. ‘La Niña’ and associated ‘blooming desert’ phenomena) [55], [56]. Such extreme and periodic precipitation events could contribute to foster episodic contacts between low- and high-elevation populations of guanaco highlighting the importance of annual rainfall at the species level and rainfall seasonality at the intraspecific level found in our models.

Based on the low affinity of guanaco to humid environments evidenced by our niche models, we may suggest that other processes could partially be responsible for the large void of guanaco occurrence in the temperate forest south of the Mediterranean area (35.3**°**–44.3**°**S). For instance, while paleontological and zooarchaeological reports that some extinct camelids may have been found in this area (i.e. *Paleolama* and/or *Hemiauchenia*), such records clearly indicate an absence of *Lama guanicoe* in this area during the end of the Pleistocene and beginning of the Holocene in spite of the existence of large open areas with dominance of grasses and Asteraceae [57], [58], confirming the expansion of guanaco from north to south during the Holocene [23]. Further south, in the continental area of the Chilean Patagonia, the recorded presence of guanaco has been explained as an introgression of peripheral populations from a dryer core environment to the east, in Argentina, into a more humid environment at the west [18]. The only extant biogeographic barriers to the south are the Magellanic Strait and the Beagle channel, established approximately 8,000 years ago [18].

We believe that our models capture essential features of guanaco’s niche, characterized mainly by climatic variables, such as annual precipitation and precipitation seasonality. However, we recognize that other factors may eventually prevent the selection of important variables [18]. For example, our niche models predict intermediate suitability in some areas in south-central Chile (i.e. mountain area of the Maule region, 35.3°S and the Alto Bio-Bio, 38.5°S) despite the absence of current sighting records or signs of guanaco presence, but historical presence some decades or centuries ago. A similar situation occurs in the central valley and coastal mountains of Chile’s Mediterranean zone, where guanacos have not been recorded in recent times [18] but, according to our models, represent suitable areas for its occurrence. This can be seen as strength of environmental niche modeling [7], [11], since recent anthropic pressures highly prevalent in central Chile [59], have prevented guanaco occurrence where suitable niche conditions are met. Yet, explanations for the lack of correlation in other areas include (1) the widespread existence of large voided areas of guanaco occurrence identified in our models as both suitable (potential lack of sampling) and non-suitable habitat for guanacos (as would be expected), and (2) the fact that anthropic influences such as hunting and poaching extend far beyond where humans live. Recognizing these elements complicates the provision of biological explanations for the lack of statistically association between direct anthropogenic variables chosen for this modeling exercise and indirect factors such as road webs, land use and/or livestock density not included here. In spite of that, it has been long recognized that local extirpations and environmental changes induced-by-humans contributed to local extinction of guanaco [60], [61]. This situation highlights the importance of explicitly considering the geographic locations of historically and/or recently extirpated populations of guanacos in future fine-tuning of niche modeling exercises.

### Conclusions and Implications for Management and Conservation

Understanding the interaction between ecology and evolution of a species is among the top endeavors in biogeography. The study of species geographic range offers an excellent opportunity not only to provide specific hints on the mechanisms generating diversity, but may – along the way - also provide spatially explicit representations of how such process may occur in time and space [62]. Here, we have not only quantitatively unveiled the factors most relevant for guanaco occurrence, but we also provide high-resolution distribution maps for the guanaco and its phylogenetic lineages. We hope that this type of information will be routinely used in the analysis, design and implementation of scientifically sound management plans for guanaco conservation at the country-level [14]. Currently, guanaco populations are protected in less than 5% of their range distribution in Chile [44]. Information presented here shows that management and conservation efforts should be geared towards Andean and Patagonian environments. Finally, the differential niche structure exhibited among lineages shown here, raises questions on the limited success of translocation experiments should we consider genetic diversity as a prime feature to conserve guanacos in southwestern South America.

## Supporting Information

Figure S1 **Map of Chile indicating the geographic distribution of guanacos at the species-level.** Grey scale indicates environmental suitability from the lowest threshold probability in white (0.274) to the highest (0.913) in black.
(TIF)

Figure S2 **Simple additive map of geographic distribution of subspecies in Chile based upon genotypes** [**23**] **.** Grey scale indicates environmental suitability from the lowest threshold probability in white (0.25) to the highest (0.617) in black.
(TIF)

Figure S3 **Map of Chile indicating the geographic distribution of the Northern subspecies** [**23**] **.** Grey scale indicates environmental suitability from the lowest threshold probability in white (0.316) to the highest (0.966) in black.
(TIF)

Figure S4 **Map of Chile indicating the geographic distribution of the Intermediate-guanaco hybrid lineage** [**23**] **.** Grey scale indicates environmental suitability from the lowest threshold probability in white (0.244) to the highest (0.936) in black.
(TIF)

Figure S5 **Map of Chile indicating the geographic distribution of the Southern subspecies** [**23**] **.** Grey scale indicates environmental suitability from the lowest threshold probability in white (0.297) to the highest (0.817) in black.
(TIF)

Table S1 **Mean and range (in parenthesis) of environmental variables selected by environmental niche model (see** **Table 1** **).**
(DOCX)

Dataset S1 **Observed guanaco points in geographic coordinates.**
(CSV)
