# Supplementary figures and images for: Unveiling Current Guanaco Distribution in Chile Based upon Niche Structure of Phylogeographic Lineages: Andean Puna to Subpolar Forests
Source: PLoS One. 2013 Nov 12;8(11):e78894. doi: 10.1371/journal.pone.0078894 (PMC3827115; doi:10.1371/journal.pone.0078894)

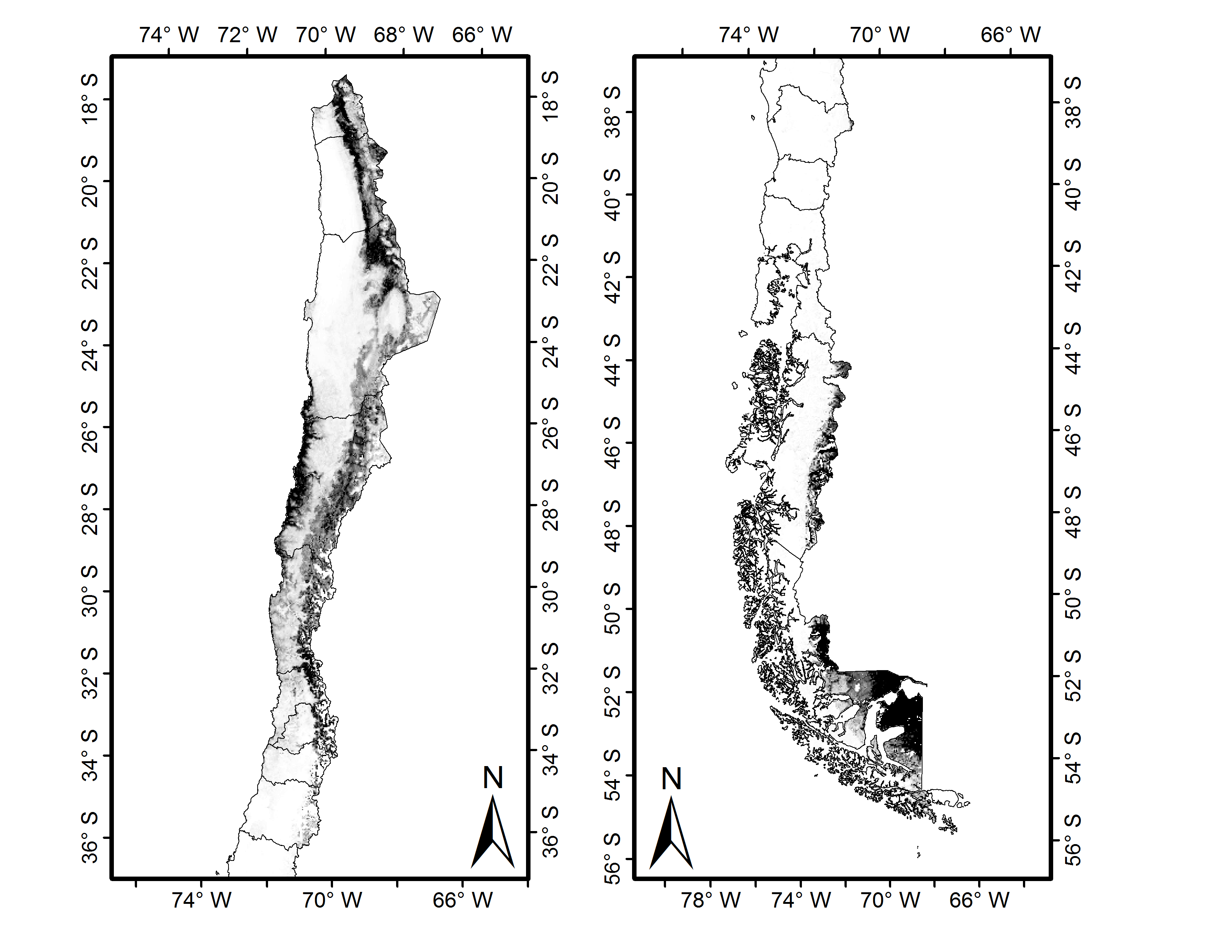

Supplement: Figure S1 — Map of Chile indicating the geographic distribution of guanacos at the species-level. Grey scale indicates environmental suitability from the lowest threshold probability in white (0.274) to the highest (0.913) in black. (TIF) [file pone.0078894.s001.tif]

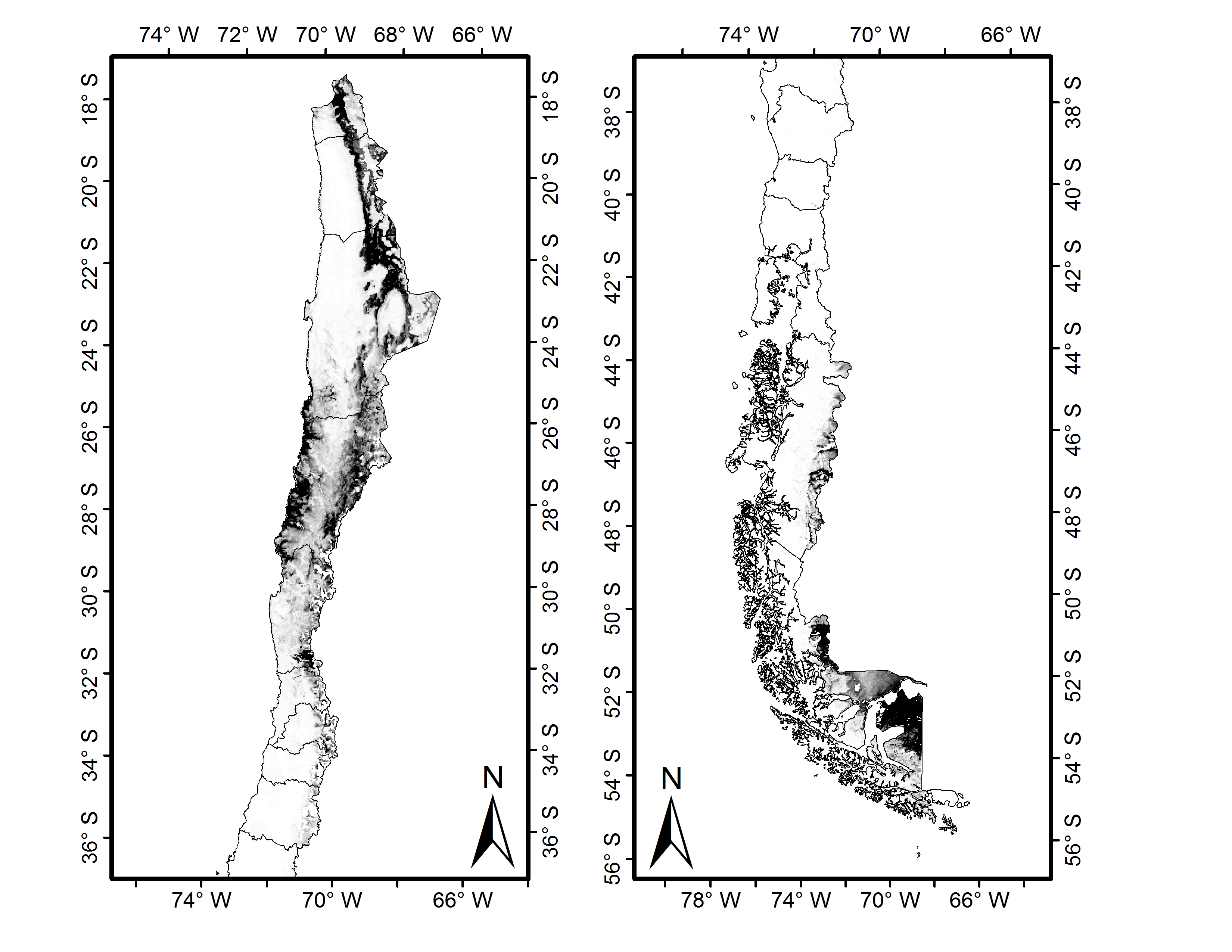

Supplement: Figure S2 — Simple additive map of geographic distribution of subspecies in Chile based upon genotypes [23] . Grey scale indicates environmental suitability from the lowest threshold probability in white (0.25) to the highest (0.617) in black. (TIF) [file pone.0078894.s002.tif]

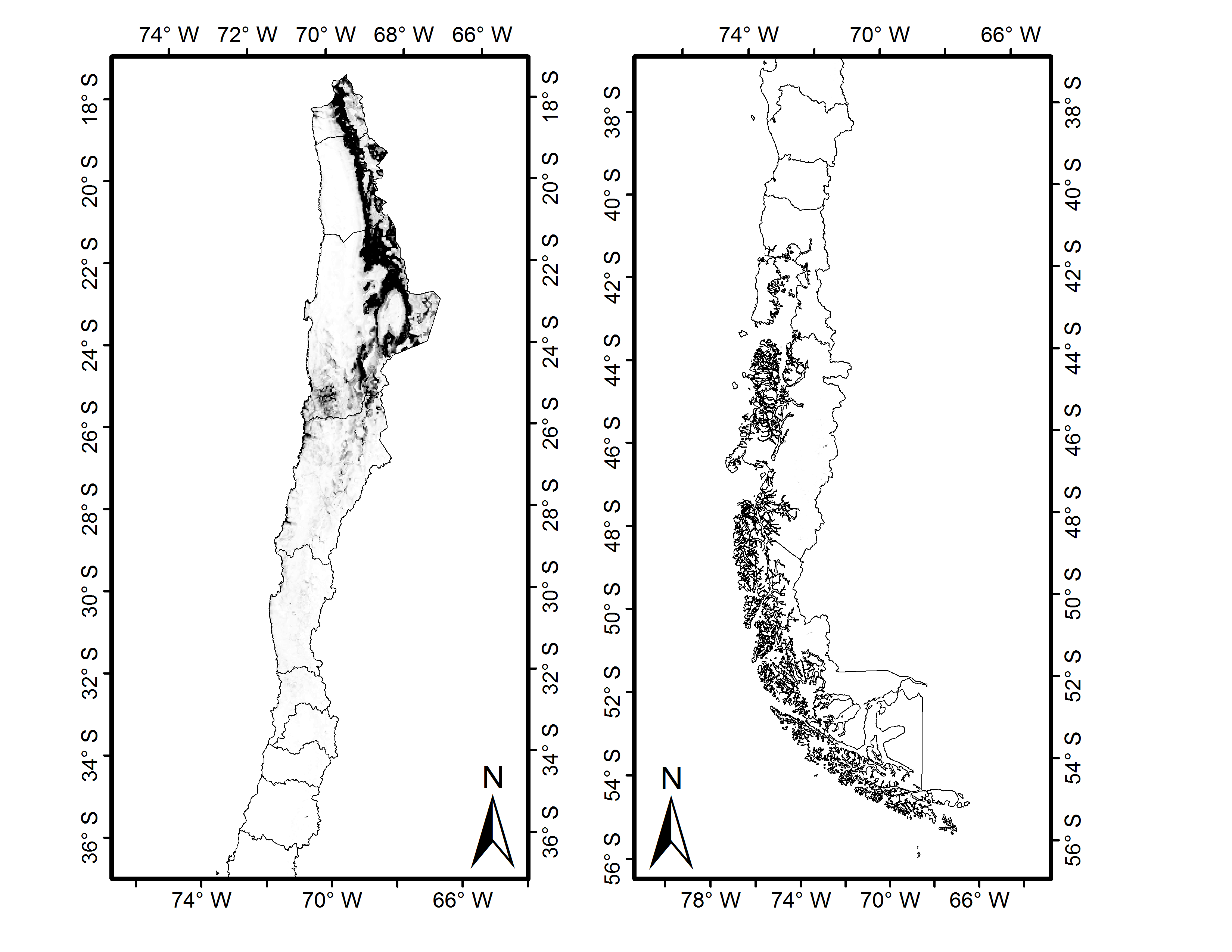

Supplement: Figure S3 — Map of Chile indicating the geographic distribution of the Northern subspecies [23] . Grey scale indicates environmental suitability from the lowest threshold probability in white (0.316) to the highest (0.966) in black. (TIF) [file pone.0078894.s003.tif]

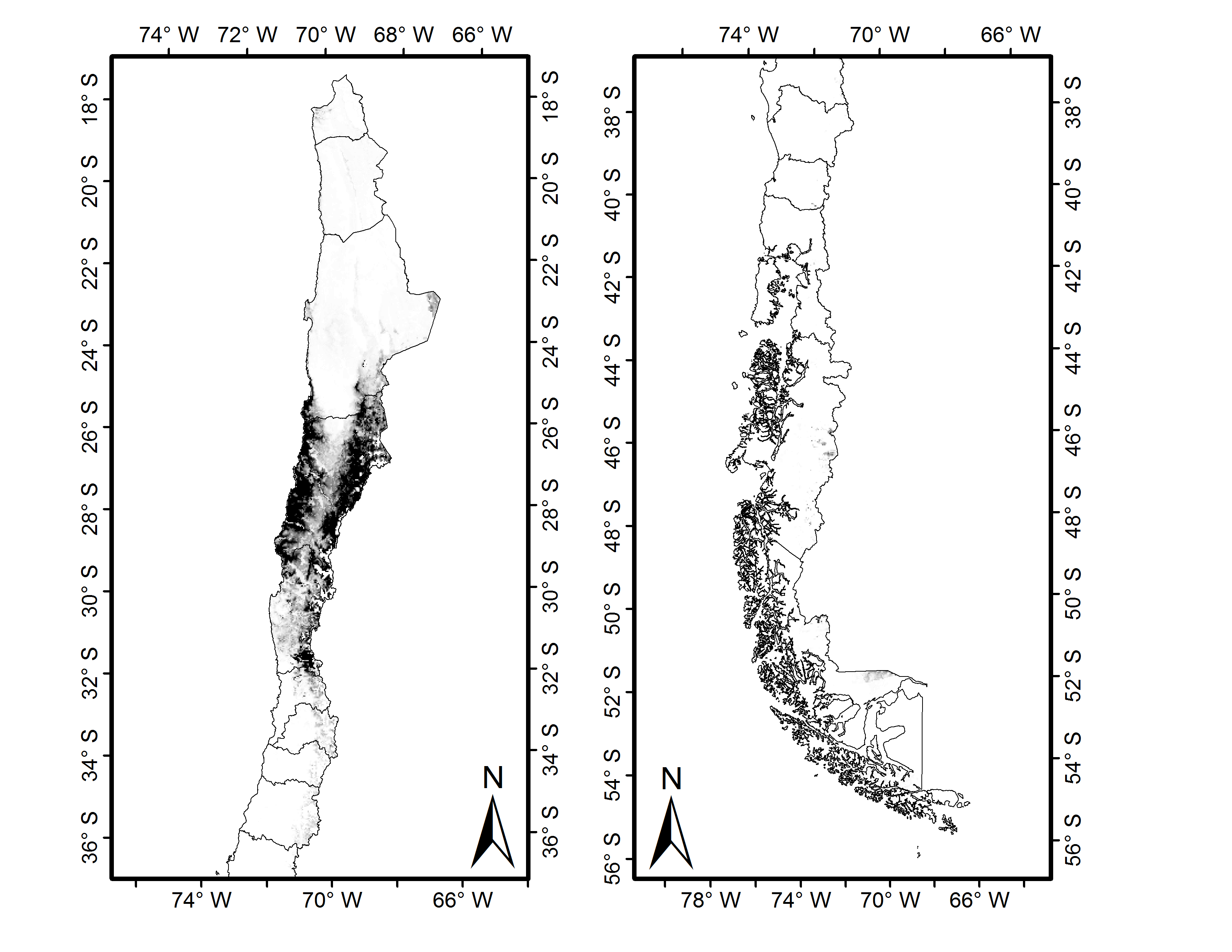

Supplement: Figure S4 — Map of Chile indicating the geographic distribution of the Intermediate-guanaco hybrid lineage [23] . Grey scale indicates environmental suitability from the lowest threshold probability in white (0.244) to the highest (0.936) in black. (TIF) [file pone.0078894.s004.tif]

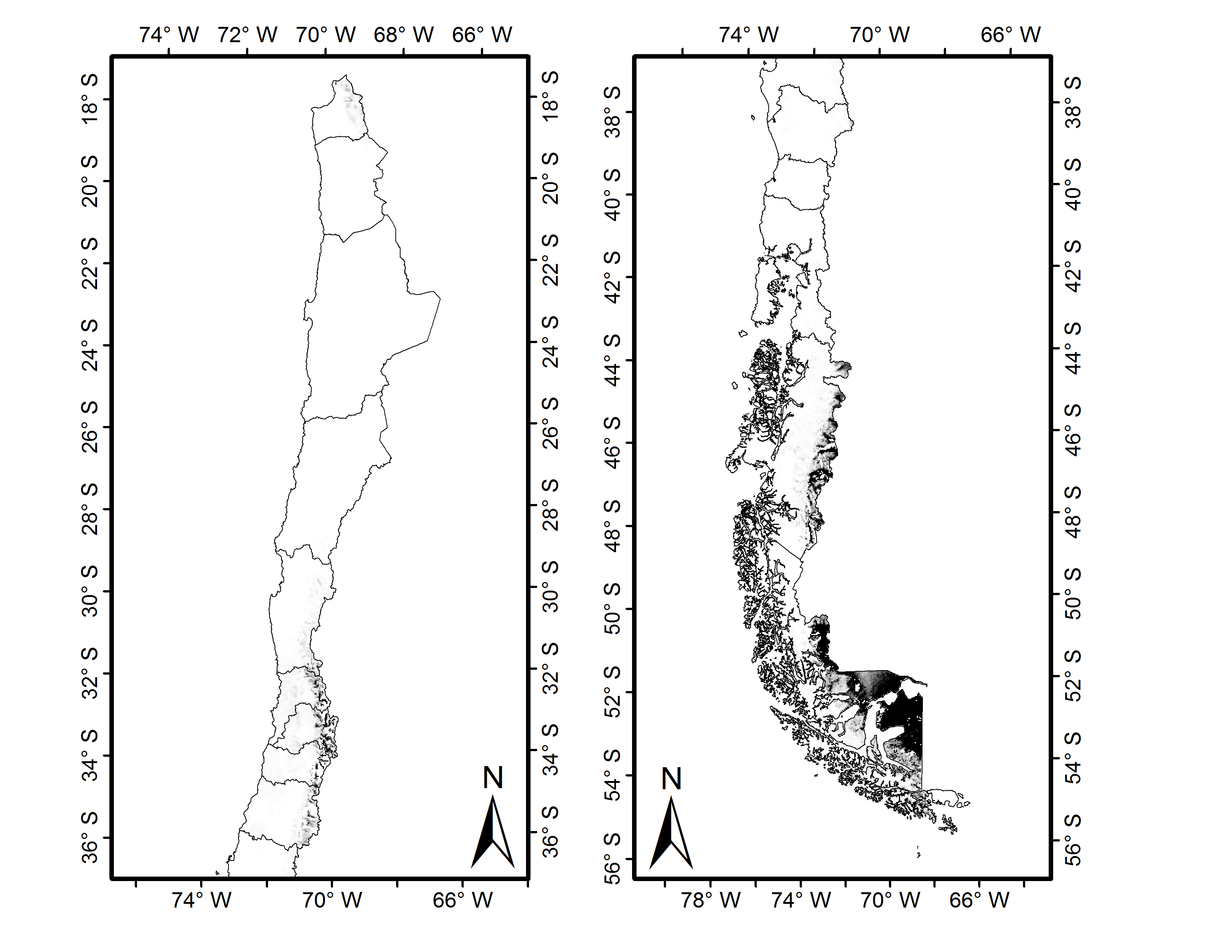

Supplement: Figure S5 — Map of Chile indicating the geographic distribution of the Southern subspecies [23] . Grey scale indicates environmental suitability from the lowest threshold probability in white (0.297) to the highest (0.817) in black. (TIF) [file pone.0078894.s005.tif]
