# Supplementary material for: Unveiling Current Guanaco Distribution in Chile Based upon Niche Structure of Phylogeographic Lineages: Andean Puna to Subpolar Forests
Source: PLoS One. 2013 Nov 12;8(11):e78894. doi: 10.1371/journal.pone.0078894 (PMC3827115; doi:10.1371/journal.pone.0078894)
Supplement: Table S1 — Mean and range (in parenthesis) of environmental variables selected by environmental niche model (see Table 1 ). (DOCX) [file pone.0078894.s006.docx]

**Table S1**.

| **Variables** |  | ***Lama guanicoe*** |  | ***L. g. cacsilensis*** (Northern subspecies) |  | **Intermediate-Hybrid lineage** |  | ***L. g. guanicoe*** (Southern subspecies) | | |  |
| --- | --- | --- | --- | --- | --- | --- | --- | --- | --- | --- | --- |
| **Bioclimatic** |  |  |  |  |  | | | |  |  | |
|  |  |  |  |  |  | | | |  |  | |
| Annual mean temperature (**°**C) |  | - |  | - | - | | | |  | 6.4 | |
|  |  |  |  |  |  | | | |  | (6.0 –8.9) | |
| Temperature seasonality (SD) |  | - |  | - | 29.3 | | | |  | - | |
|  |  |  |  |  | (8.5 – 36.0) | | | |  |  | |
| Average annual min temperatures (**°**C) |  | 1.7 |  | - | - | | | |  | - | |
|  |  | (-10.0 – 14.0) |  |  |  | | | |  |  | |
| Average annual max temperatures (**°**C) |  | - |  | 16.0 | - | | | |  | - | |
|  |  |  |  | (12.0 – 24.0) |  | | | |  |  | |
| Annual precipitation (mm) |  | 442 |  | - | 139 | | | |  | 553 | |
|  |  | (8 – 901) |  |  | (10 – 261) | | | |  | (84 – 901) | |
| Precipitation seasonality (CV) |  | - |  | 161 | 87.9 | | | |  | 21.1 | |
|  |  |  |  | (52 – 197) | (37.0 – 123.0) | | | |  | (6.0 – 94.0) | |
| **Topographic** |  |  |  |  |  | | | |  |  | |
|  |  |  |  |  |  | | | |  |  | |
| Altitude (m) |  | 874 |  | 3,413 | - | | | |  | - | |
|  |  | (6 – 4,831) |  | (67 – 4,393) |  | | | |  |  | |
| **Vegetation** |  |  |  |  |  | | | |  |  | |
|  |  |  |  |  |  | | | |  |  | |
| EVI (Enhanced vegetation Index) |  | - |  | - | 0.054 | | | |  | - | |
|  |  |  |  |  | (0.010 – 0.151) | | | |  |  | |
| Grass cover (%) |  | 6.4 |  | - | - | | | |  | 8.2 | |
|  |  | (0.0 – 11.0) |  |  |  | | | |  | (0.0 – 11.0) | |
